# Co-inoculation of endophytic bacteria enhances hydroponic wheat performance and zinc biofortification through root exudates modulation

**DOI:** 10.3389/fmicb.2026.1844753

**Published:** 2026-07-10

**Authors:** Nazish Jabeen Abbasi, Ancao Pan, Jazbia Shirin, Qizhen Liu, Ivan Mustać, Gabrijel Ondrasek, Muhammad Shafiq Shahid, Yasir Hamid, Ying Feng

**Affiliations:** 1 College of Environmental and Resource Sciences, Zhejiang University, Hangzhou, China; 2 School of Environment and Energy, Peking University Shenzhen Graduate School, Shenzhen, China; 3 Faculty of Agriculture, The University of Zagreb, Zagreb, Croatia; 4 Department of Plant Sciences, College of Agricultural and Marine Sciences, Sultan Qaboos University, Muscat, Oman

**Keywords:** hyperaccumulator, metabolic reprogramming, metabolomics, root exudates, SynCom, zinc

## Abstract

**Purpose:**

Microbe-assisted zinc (Zn) biofortification offers a sustainable strategy for enhancing wheat productivity and nutritional value. This study evaluated whether two endophytic bacteria from the Zn hyperaccumulator *Sedum alfredii*, applied individually or as a SynCom, could improve wheat growth, Zn uptake, physiological performance, and root exudate modulation under hydroponic conditions.

**Methods:**

Wheat seedlings were grown under hydroponic conditions and inoculated with SaPA1 and SaBR2 individually or as a consortium, and their impacts on growth, Zn uptake, physiological characteristics, root morphology, and root exudate metabolomics were assessed after 30 days.

**Results:**

All inoculated treatments improved Zn uptake, wheat growth, plant height, and leaf photosynthetic performance relative to the control, with the SynCom showing the strongest overall effect. Zn concentrations increased by 42.49% in roots and 46.6% in shoots under the consortium treatment compared to the control. Zn accumulation was also significantly enhanced by endophytic inoculation, with the consortium producing the greatest increase, reaching about 6-fold in roots and nearly 3-fold in shoots relative to the control. Additionally, non-targeted LC–MS profiling revealed clear treatment-dependent shifts in root exudates, with the SynCom showing the strongest metabolic reprogramming and greater organic acid exudation.

**Conclusion:**

These findings show that endophytic bacteria derived from a Zn hyperaccumulator can improve wheat growth, Zn uptake, physiological performance, and root exudate remodeling under hydroponic conditions. Overall, the results support the potential of endophytic bacteria, especially as a SynCom, as a microbial strategy for Zn biofortification in wheat.

## 1 Introduction

Zinc (Zn) is an essential micronutrient for plant growth, which plays a critical role in photosynthesis, cell wall formation, gene regulation, and stress tolerance (Shakeel et al., 2024). In higher plants, approximately 3,000 proteins depend on Zn prosthetic groups for structural integrity. Zn deficiency reduces plant growth, yield, and the nutritional quality of edible parts. Zn is also essential for human health, where it supports growth, immunological function, and several physiological processes. It functions as a neuromodulator within the central nervous system and has gained potential supportive treatment in COVID-19 therapy (Goldberg and Lippard, 2018; Skalny et al., 2020). Zn deficiency is a major global health challenge, especially in developing countries. According to the World Health Organization (WHO), estimates it affects nearly 2 billion people world-wide, predominantly in Asia and Sub-Saharan Africa (Singh et al., 2023; Hussain et al., 2022). Additionally, Zn insufficiency disproportionately impacts vulnerable groups, particularly pregnant women and children, where 82% of pregnant women globally consume inadequate Zn, often resulting in fetal growth abnormalities (Wang et al., 2020). Despite established dietary recommendations, the global Zn insufficiency persists as a major nutritional challenge (Wang et al., 2020).

Wheat (*Triticum aestivum* L.) is one of the worlds’ most important cereal crop, cultivated on over 240 million hectares globally, with an annual production of over 799 million tonnes (FAO, 2024). It serves as a primary food source for around 40% of the global population (Giraldo et al., 2019) and provide roughly 70% of daily calorie consumption in many South Asian nations, including Nepal, Pakistan, India, and Bangladesh (Singh et al., 2023). Zn inadequacy in wheat is a serious concern, especially in addressing ‘hidden hunger,’ where micronutrient shortages significantly impact the human health, particularly in women and children (Biesalski, 2021; Abbaspour et al., 2014). Despite being a staple diet, wheat naturally contain low Zn quantity, typically ranging from 20 to 35 mg kg^−1^, which is lower to meet daily Zn requirements. Therefore, biofortification of wheat with Zn is imperative to reduce the risk of Zn deficiency in people (Khalid et al., 2022). Current recommendations suggest that Zn content in wheat should be elevated to 45 mg kg^−1^ to adequately fulfill daily Zn requirements. To overcome this issue, several innovative strategies for biofortifying wheat with Zn have been implemented (Yadav et al., 2023). One conventional approach involves the utilization of chemical fertilizers to directly supply micronutrients to plants. However, this method has some drawbacks, including the low absorption (2–4%) of applied Zn (Singh et al., 2017), negative environmental consequences associated with chemical use, such as disrupting nitrogen balance and reducing soil fertility (Lockhart et al., 2013; Wu and Ma, 2015). Excessive application of Zn fertilizer may lead to resource inefficiency and contribute to soil contamination (Liao et al., 2025). Furthermore, repeated high-rate Zn application may elevate heavy metal deposition in soil, thereby posing a possible hazard to soil ecology and functionality (Dai et al., 2023).

Microbial-mediated Zn biofortification has emerged as a promising alternative, offering both economic benefits and potential environmental sustainability. This approach is particularly effective in enhancing Zn bioavailability and accumulation in wheat grains, which is essential for addressing worldwide Zn nutrition and health challenges (Mahmud et al., 2021). Zn-solubilizing bacteria (ZSB) possess the ability to convert inorganic Zn into bioavailable forms. Bacteria that solubilize Zn compounds achieve this by producing and excreting organic acids in the soil that acidify the surrounding environment, lowering the soil pH, chelate Zn cations, and mobilize available Zn fractions, thereby increasing Zn availability at the root interface (Rasul et al., 2019). As a result, ZSB can improve Zn intake, plant nutrition, and wheat growth. Endophytic Zn-solubilizing bacteria further contribute to this process by colonizing internal plant tissues without causing damage, which may strengthen root-associated Zn mobilization and uptake and improve Zn biofortification efficiency in wheat. These bacteria enhance plant development, nutrient absorption, and Zn levels in wheat by increasing Zn solubility and availability in the soil. These advantages are significantly associated with root exudation, which can alter rhizosphere chemistry and affect Zn bioavailability at the root interface. Root exudates comprise a complex combination of low-molecular-weight chemicals emitted by plant roots, crucial for influencing rhizosphere chemistry. These exudates can chelate metals and modify their solubility, thereby directly affecting the availability of Zn at the root–solution contact (Chen et al., 2014). It is closely connected to plant–microbe interactions, as exudates facilitate the recruitment and sustenance of rhizosphere microbes, while microbial activity can reciprocally influence exudation patterns, collectively impacting nutrient acquisition and stress responses.

SaPA1 (*Pantoea agglomerans*) and SaBR2 (*Brevibacterium epidermidis*), identified from *Sedum alfredii*, have been previously reported to enhance wheat growth and Zn absorption in varying Zn concentration soils (Li et al., 2025). We hypothesized that endophytic strains (SaPA1, SaBR2, and SaPA1 + SaBR2) isolated from the Zn hyperaccumulator *Sedum alfredii* would successfully colonize wheat roots under hydroponic conditions, improve root morphology, plant growth, and enhance Zn uptake in both the roots and shoots, and modulate root exudate profiles. This study aimed to: (1) evaluate the effects of SaPA1, SaBR2, and their SynCom on wheat growth, root morphology, and Zn uptake under hydroponic conditions; (2) assess the impact of bacterial inoculation on photosynthetic performance; and (3) ascertain whether inoculation modifies root exudate profiles.

## 2 Materials and methods

### 2.1 Experimental setup and growth conditions

A nutrient-solution experiment was conducted in the growth chamber laboratory at the College of Environmental and Resource Sciences, Zhejiang University, from November to December 2025, using wheat (*Triticum aestivum* L.) cv. Jinchun 6 (津春6号; JINCHUN6HAO) as the test crop. Seeds were obtained from Hebei Fuyichun Seed Co., Ltd. visually healthy and uniform seeds were selected for germination. A hydroponic system was used to provide tightly controlled growth conditions and to reduce the impact of soil heterogeneity on treatment outcomes. Seeds were surface sterilized and germinated at 30 °C in darkness, after which uniform seedlings were promptly transferred to opaque 1.5-L black plastic pots filled with Hoagland’s nutritional solution. Seedlings were supported in a perforated lid using sponge plugs; each pot had a plastic foam cover with four small holes, and four seedlings were fixed with cotton in each hole (16 seedlings/pot) to maintain uniform plant density across treatments. The nutrient solution was replaced every three days and adjusted to pH 5.5 ± 0.1 every day. Plants were grown in a growth chamber under a photosynthetic photon flux density of 300 μmol m^−2^ s^−1^, a light/dark photoperiod of 10/14 h, day/night temperatures of 25/20 °C, and day/night relative humidity levels of 70%/85%.

### 2.2 Inoculation with endophytic bacteria under hydroponic conditions

Two endophytic bacterial strains, SaPA1 (*Pantoea agglomerans*) and SaBR2 (*Brevibacterium epidermidis*), previously isolated from the Zn hyperaccumulator *Sedum alfredii*, were used in this hydroponic experiment (Li et al., 2025). Each strain was cultured separately in liquid Luria–Bertani (LB) medium for 48 h at 30 °C on a rotary shaker. Cells were collected by centrifugation, rinsed twice with phosphate-buffered saline (PBS), and suspended in sterile physiological saline (0.85% NaCl). The inoculum density was calibrated to 10^8^ CFU mL^−1^, and the resulting suspensions were used for inoculation. For bacterial treatments, 5 mL of inoculum was added to 1.5 L of nutrient solution using a pipette, ensuring no contact with above-ground tissues to reduce contamination; the control received Hoagland solution without bacterial solution. The experiment was performed in a growth chamber, and treatments were randomly assigned with three replications. Plants were harvested at 30 days post-inoculation; shoots and roots were separated, fresh biomass was recorded, and samples were oven-dried to a consistent weight for further analysis.

### 2.3 Assessment of root morphological parameters

At the end of the one-month hydroponic experiment, roots were carefully harvested and rinsed three times with distilled water to remove any remaining nutrient solution. The intact root systems were initially captured with a digital camera and then scanned under standardized conditions (EPSON scanner, Professional Mode, 600 dpi, 48-bit full color). The scanned images were analyzed in WinRHIZO using a consistent procedure to measure root morphological characteristics.

### 2.4 Photosynthetic parameters

The net photosynthetic rate (AN_NN), stomatal conductance (gsw), intercellular CO_2_ concentration (Ci_ii), and transpiration rate (Emm_E) were quantified on completely expanded leaves using a portable photosynthesis system (LI-6400XT, LI-COR Biosciences, United States) fitted with a red–blue LED leaf chamber. Measurements were conducted at a photosynthetic photon flux density of 300 μmol m^−2^ s^−1^. During measurements, the reference CO_2_ concentration was kept at 380 ± 5 μmol mol^−1^, the chamber airflow rate was adjusted to 500 μmol s^−1^, and leaf temperature was regulated at 25 °C. The selected leaf from each plant was secured in the chamber and allowed to stabilize before the recording of steady-state measurements. Measurement was conducted in the morning between 8:00 and 10:00 a.m., on three selected fully expanded leaves analyzed per treatment, to avoid possible stomatal closure during the middle of the day (Benhmimou et al., 2018).

### 2.5 Biomass assessment and mineral element analysis

At the end of the experiment, shoots and roots were separated and weighed to determine fresh biomass. Roots were thoroughly cleansed to eliminate externally adsorbed ions and then rinsed with deionized water. Plant tissues were dried at 65 °C; until achieving a constant weight and the dry weights of shoots and roots were recorded for biomass determination and Zn accumulation calculations. Then dried shoot and root samples were then finely ground and subjected to acid digestion with 5 mL of HNO₃ and 1 mL of H₂O₂. After complete digestion, the solution was diluted to 40 mL with deionized water and subsequently filtered through a 0.22 μm cellulose acetate membrane before elemental analysis. Zn concentrations in shoot and root digests were measured via ICP–MS, employing a standard Zn solution for instrument calibration and incorporating routine blanks for quality assurance.

### 2.6 Collection of root exudates and examination

Root exudates were obtained according to the method of Chen et al. (2014) where intact plants were carefully extracted from the hydroponic systems and the roots were placed in 100 mL of 0.5 mM CaCl₂ for 4 h. The obtained solution was subsequently purified by passing through [Amberlite IR-120 (H^+^ form)] and then through another resin column [Dowex 1 × 8,100–200 mesh (Cl^−^ form)]. The exudate was dried using a rotary evaporator and reconstituted in methanol for subsequent LC–MS analysis (Agilent 1,200 series). Sample extraction and instrumental analysis for non-targeted metabolite profiling were conducted at a commercial metabolomics lab. Samples were kept at −80 °C until analysis. For extraction, 4 mL of each sample was thawed on ice and lyophilized; the residue was reconstituted with 100 μL of water and extracted with 400 μL of pre-chilled (−40 °C) methanol: acetonitrile (1:1, v/v) containing internal standards. This was followed by vortexing, ultrasonication in an ice-water bath, incubation at (−40 °C) for protein precipitation, and centrifugation at 12,000 rpm for 15 min at 4 °C. The supernatant was allocated for LC–MS analysis, and a pooled quality-control (QC) sample was created by combining equal aliquots from the samples. Chromatographic separation was conducted on a Vanquish UHPLC utilizing a Phenomenex Kinetex C18 column, employing water with 0.01% acetic acid as mobile phase A and a mixture of isopropanol and acetonitrile (1:1, v/v) as mobile phase B (autosampler maintained at 4 °C, injection volume of 2 μL). Mass spectra were obtained using an Orbitrap Exploris 120 in both positive and negative ionization modes, employing data-dependent MS/MS acquisition (full MS resolution 60,000; MS/MS resolution 15,000; stepwise collision energy 20/30/40). Metabolomics Standards Initiative (MSI) framework, where Level 1 indicates matching of MS1, MS2, and retention time with authentic standards, Level 2 denotes alignment with public databases, Level 3 indicates putative compound-level annotation, and Level 4 represents unknown compounds.

### 2.7 Statistical analysis

Statistical analysis was conducted using SPSS 25.0 (IBM Corp., USA) and Minitab 18. The data were provided as mean ± standard deviation (SD) with three biological replicates for each treatment. Plant variables were analyzed using one-way ANOVA at *p* < 0.05. Upon ANOVA revealing significant treatment effects, Tukey’s test was used for pairwise comparisons across all treatments, while Dunnett’s test was used to compare each inoculated treatment with the control. Alphabet letters denoted statistically significant differences between the treatment group and the control group, indicating *p* < 0.05, respectively. The graphical work was performed using GraphPad Prism 8 (GraphPad Software Inc., United States) and Origin Pro (Graphics and Analysis). Raw files of root exudates were converted into mzXML format using ProteoWizard, and metabolites were annotated using proprietary and plant-specific databases. For metabolomics, raw data were preprocessed by the service provider via RSD-based noise filtering, missing-value filtering, half-minimum imputation, and TIC normalization. PCA was performed following log transformation and centering, while OPLS-DA was executed after log transformation and UV scaling. The robustness of the model was evaluated using 7-fold cross-validation and 200 permutation tests. Differential metabolites were screened based on variable importance in projection (VIP) > 1 from the OPLS-DA model and *p* < 0.05 from Student’s *t*-test. Q-values were generated in the metabolomics output, and adjusted statistics, including Holm and FDR adjustments, were presented in subsequent pathway-level studies.

## 3 Results

### 3.1 Enhancement in root and shoot fresh biomass and plant height

Bacterial inoculation markedly enhanced the shoot and root fresh biomass (*p* < 0.05). It was noted that SaPA1 + SaBR2 consortia yielded the highest shoot and root fresh weights (36.46 g and 15.98 g, respectively) in contrast to the control (20.61 g and 2.24 g). In addition, single-strain inoculation also enhanced the biomass, with shoot fresh weight reaching 24.58 g for SaPA1 and 29.21 g for SaBR2, while root fresh weight increased to 10.15 g and 10.41 g, respectively.

Plant height also exhibited a consistent rise from 5 to 30 days across all treatments, with distinct differences among inoculation protocols. The consortium (SaPA1 + SaBR2) yielded the tallest plants, growing from 14 to 54 cm over the experimental period, followed by SaBR2 (12–49 cm) and SaPA1 (10–45 cm), while CK remained the shortest at 8–43 cm. The differences in treatment effects increased as the experiment progressed, ending in the most significant height advantage at 30 days.

The inoculation of endophytic bacteria markedly enhanced the wheat root morphology. The consortium treatment (SaPA1 + SaBR2) exhibited the most pronounced response, producing the longest total root length (29.5 cm plant^−1^) and the largest root surface area (1263.48 cm^2^ plant^−1^) in comparison to the control (18.53 cm plant^−1^; 807.46 cm^2^ plant^−1^) and the individual treatments. SaPA1 (19.03 cm plant^−1^; 989.53 ± 5.8 cm^2^ plant^−1^) and SaBR2 (22.03 cm plant^−1^; 1121.32 cm^2^ plant^−1^) (Table 1).

**Table 1:** Root parameters and main interactive effect of bacterial consortium on total root length and root surface area of wheat.

Treatments	Total root length (cm plant^−1^)	Surface area (cm^2^ plant^−1^)
Control	18.53 ± 1.86 c	807.46 ± 8.75 d
SaPA1	19.03 ± 3.13 c	989.53 ± 5.8 c
SaBR2	22.03 ± 2.25b	1121.32 ± 8 b
SaPA1 + SaBR2	29.5 ± 3.49 a	1263.48 ± 7.31 a

Values are presented as mean ± SD. Different lowercase letters within a column indicate significant differences among treatments at *p* < 0.05.

### 3.2 Zn concentration in roots and shoots

**Figure 1:**
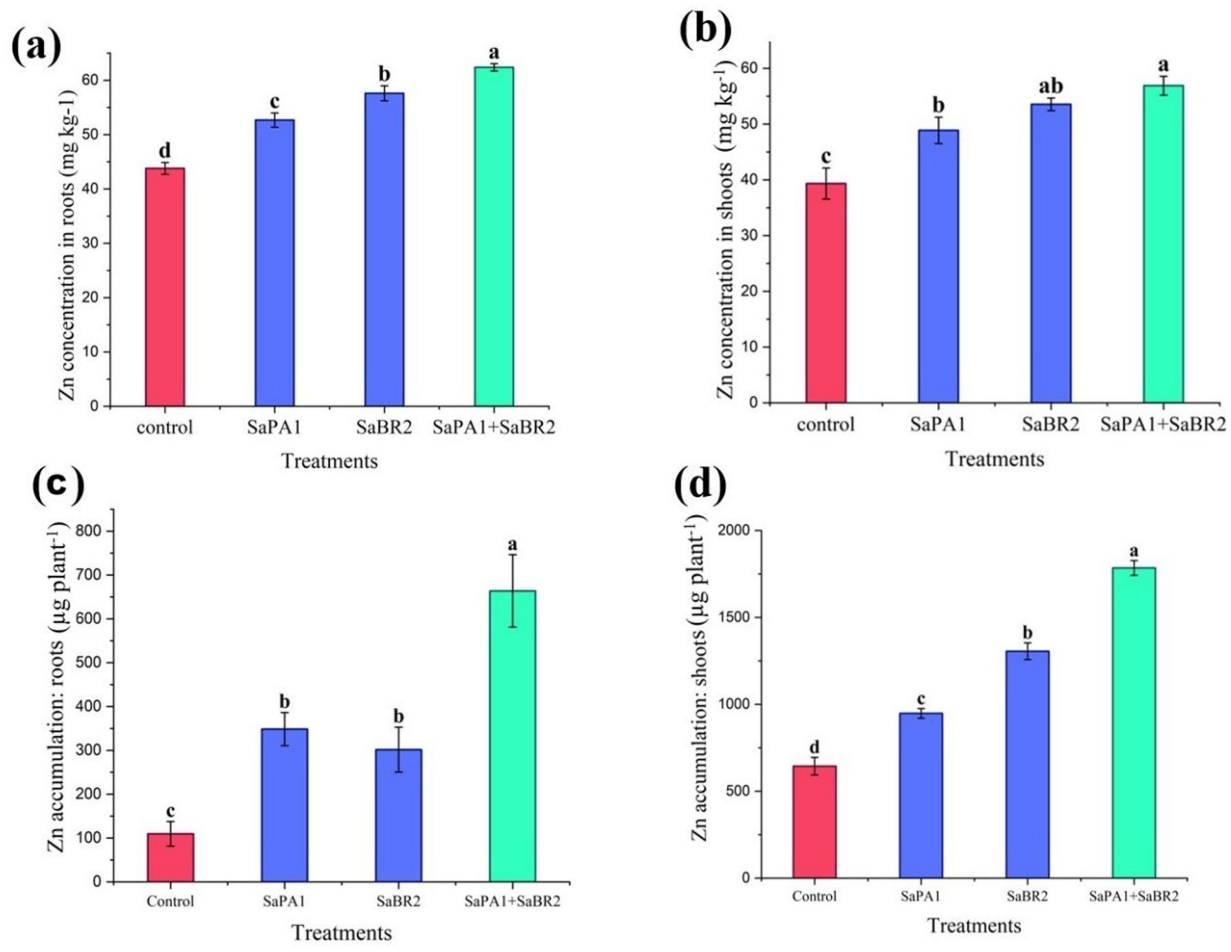
Effects of endophytic bacterial inoculations on zinc concentration and accumulation in wheat. **(a)** Zn concentration in roots and **(b)** Zn concentration in shoots, **(c)** Zn accumulation in roots, and **(d)** Zn accumulation in shoot under different treatments. Different lowercase letters on the column indicate significant differences among treatments at *p* < 0.05.

Endophytic bacterial inoculation markedly increased the Zn concentration in both roots and shoots (*p* < 0.05). Compared to the control, SaPA1 increased the Zn levels by 20.31% (root) and 24.27% (shoot), whereas SaBR2 enhanced it by 31.56% (root) and 33.77% (shoot). The consortium (SaPA1 + SaBR2) achieved the highest improvement, increasing Zn concentration by 42.49% in roots and 46.66% in shoots relative to the control group (Figures 1a,b). In particular, Zn accumulation in the consortium treatment increased by almost six-fold in roots and nearly three-fold in shoots compared with the control, suggesting a synergistic effect of the two strains (Figures 1c,d).

### 3.3 Impact of endophytic inoculation on leaf gas exchange metrics

It was observed that transpiration rate (E) differed among treatments. The control showed an average of 3.2077 mmol H₂O m^−2^ s^−1^, SaPA1 3.2315 mmol H₂O m^−2^ s^−1^, and SaBR2 showed a higher value of 4.7024 mmol H₂O m^−2^ s^−1^, while the consortium (SaPA1 + SaBR2) recorded the highest value at 5.083 mmol H₂O m^−2^ s^−1^. Stomatal conductance (gsw) also varied across treatments. CK had the lowest value 0.217 mol H₂O m^−2^ s^−1^, whereas SaPA1 and SaBR2 increased to 0.36155 and 0.2537 mol H₂O m^−2^ s^−1^, respectively. The consortium showed the highest gsw values 0.37607 mol H₂O m^−2^ s^−1^. Net photosynthetic rate (A) showed treatment-dependent differences. The control showed the value of 14.7601 μmol CO₂ m^−2^ s^−1^, while SaPA1, SaBR2, and SaPA1 + SaBR2 recorded 13.2234, 16.593, and 17.485 μmol CO₂ m^−2^ s^−1^, respectively. Intercellular CO_2_ concentration (Ci) increased progressively across treatments. The highest concentration was observed in the SaPA1 + SaBR2 treatment (325.451 μmol mol^−1^), followed by SaBR2 (314.745 μmol mol^−1^). and SaPA1 (306.136 μmol mol^−1^). Overall, all photosynthetic parameters (E, gsw, A, and Ci) differed significantly among treatments at *p* < 0.05.

### 3.4 Bacterial inoculation transforms wheat root exudates metabolites

**Figure 2:**
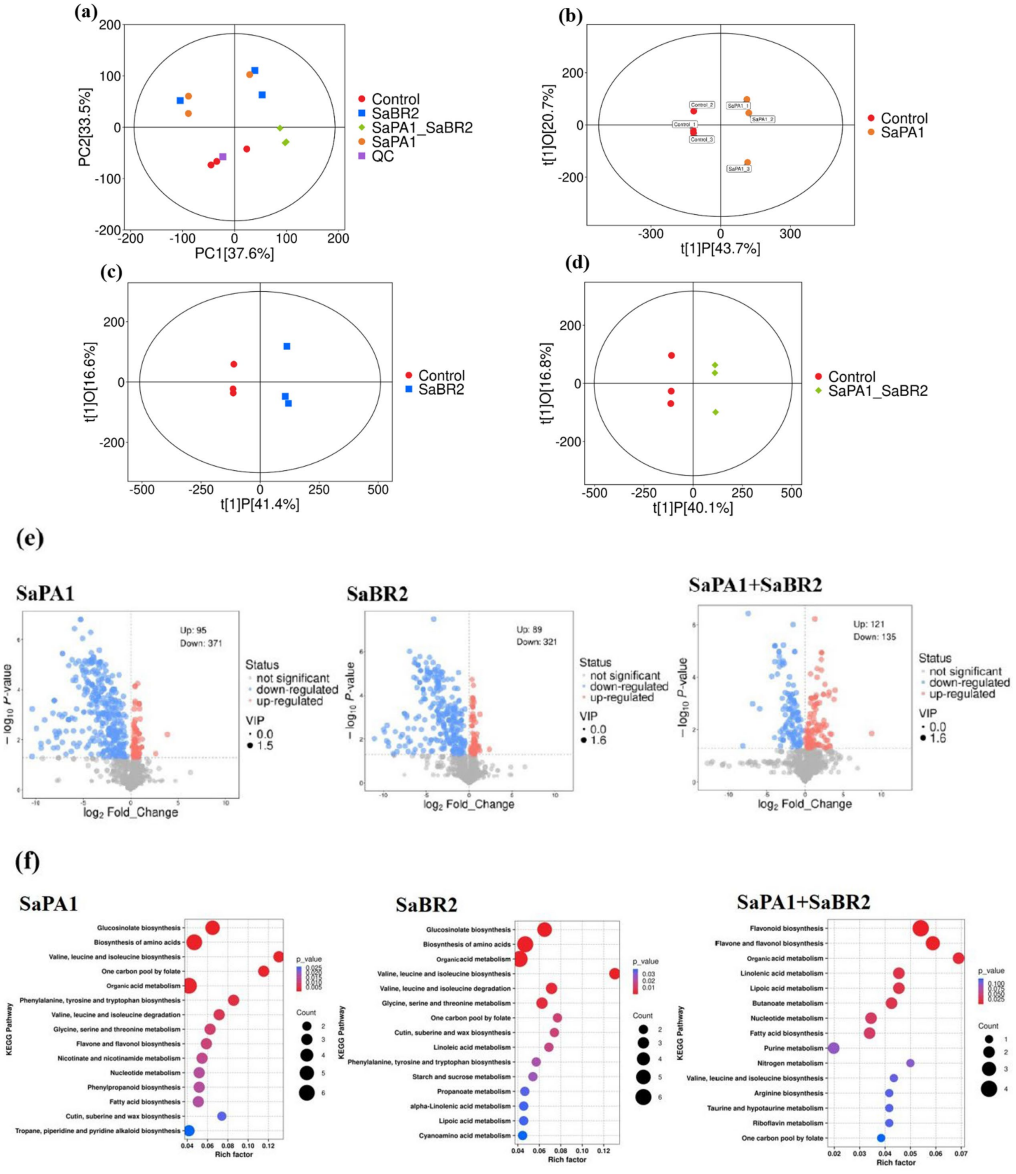
Multivariate and differential metabolite analyses of wheat root exudates under different bacterial treatments: **(a)** PCA score plot, **(b)** OPLS-DA score plot for Control vs. *Sa*PA1, **(c)** OPLS-DA score plot for Control vs. *Sa*BR2, **(d)** OPLS-DA score plot for Control vs. *Sa*PA1 + *Sa*BR2, **(e)** volcano plots of differential metabolites, and **(f)** KEGG pathway enrichment analysis of differential metabolites.

Non-targeted LC–MS profiling of wheat root exudates identified 37,248 metabolic features in total across all the samples. PCA demonstrated close clustering of QC injections and consistent performance throughout the analytical process, indicating high reproducibility and minimal drift (Figure 2). Biological replicates were grouped consistently, and treatment groups exhibited distinct separation in PCA space (Figure 2a). OPLS-DA score plots further indicated clear separation of SaPA1, SaBR2, and SaPA1 + SaBR2 samples from the control, confirming treatment-dependent shifts of the root exudate metabolites (Figures 2b–d). The OPLS-DA models demonstrated robustness, evidenced by good explanatory and predictive values (R^2^Y = 0.999–1.000; *Q*^2^ = 0.910–0.939), with permutation testing further validating model reliability. Differential feature analysis revealed 11,775 modified features in SaPA1 (4,436 up; 7,339 down), 9,967 in SaBR2 (3,979 up; 5,988 down), and 6,826 in the SaPA1 + SaBR2 (3,681 up; 3,145 down). Volcano plots revealed significantly changed metabolites between each inoculation treatment and the control (*p* < 0.05) (Figure 2e). Differential metabolites were identified using a combined criterion of VIP > 1 from the OPLS-DA model and a Student’s *t*-test (*p* < 0.05). KEGG enrichment analysis showed significant pathway-level shifts after inoculation (*p* < 0.05) (Figure 2f). Across treatments, up-regulated features were mostly enriched in lipid-related pathways (fatty acid biosynthesis and associated processes), while down-regulated features were typically associated with amino acid and carbohydrate metabolism (including amino acid biosynthesis and branched-chain amino acid pathways). The consortium integrated a balanced differential profile with enrichment of secondary metabolism pathways (including flavonoid and flavone/flavonol-related pathways), facilitating targeted pathway-level remodeling of root exudation instead of diffuse changes (Figure 2f).

Root exudates from wheat plants subjected to various bacterial treatments (Control, SaPA1, SaBR2, SaPA1 + SaBR2) were examined for organic acid. Overall, organic acid concentrations elevated with bacterial inoculation. Oxalic acid exudation was 90% more in the SaPA1 + SaBR2 consortium treatment (86.87 mg/kg h FW) than in the control (45.77 mg/kg h FW). The exudation of citric acid also increased, with the consortium treatment (30.86 mg/kg h FW) exhibiting a 111% elevation compared to the control (14.55 mg/kg h FW). Malic acid and tartaric acid increased by 40–50% in the SaPA1 + SaBR2 treatment, yielding values of 45.11 mg/kg h FW and 47.56 mg/kg h FW, respectively, in contrast to the control values of 32.91 mg/kg h FW and 16.74 mg/kg h FW. Additionally, pyrogulamate exudation was seen in the consortium treatment (32.36 mg/kg h FW) in contrast to the control (16.25 mg/kg h FW).

## 4 Discussion

### 4.1 Effects of endophytic inoculation on plant growth root architecture

**Figure 3:**
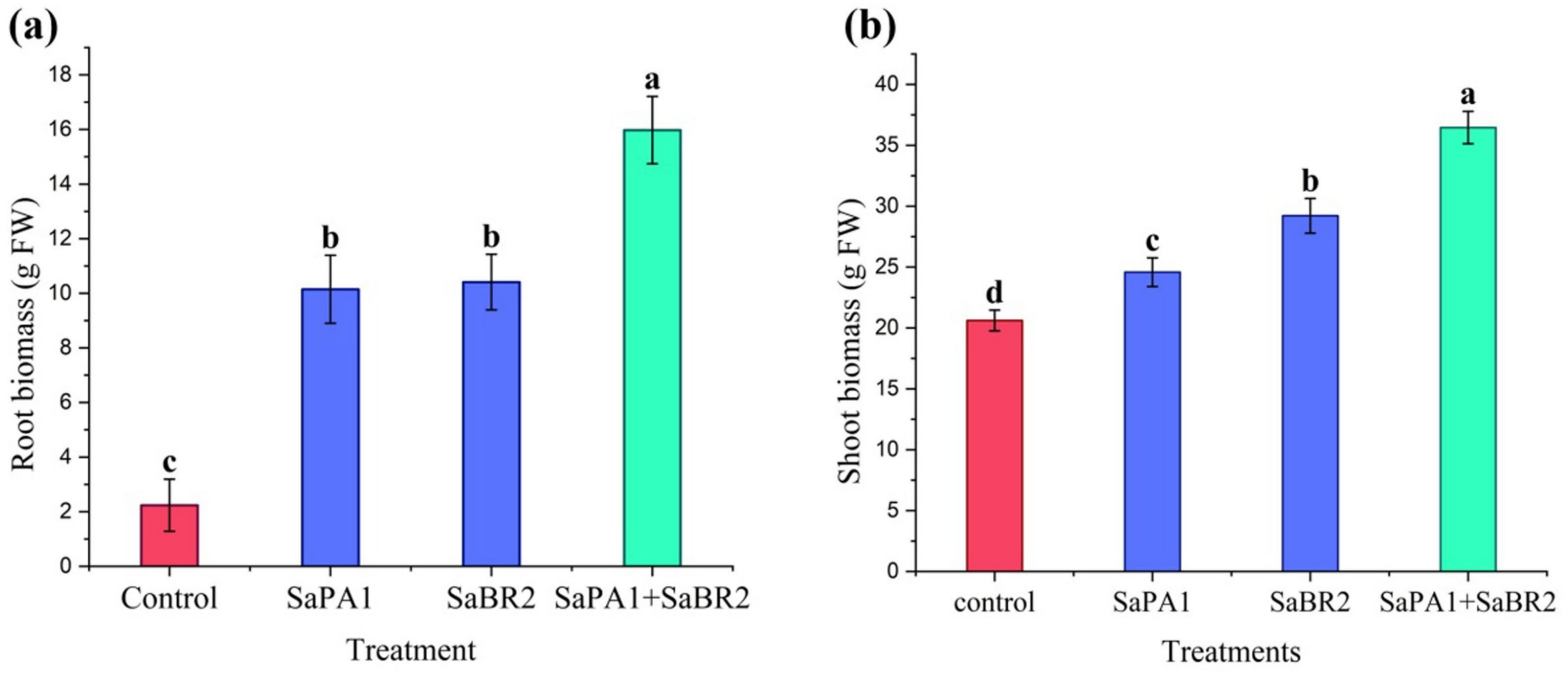
Effects of bacterial inoculation on **(a)** root fresh biomass and **(b)** shoot fresh biomass in wheat. Error bars denote standard deviation and statistical significance, as shown using ANOVA (*p* < 0.05).

Root and shoot biomass increased across all bacterial treatments relative to the control, with the greatest increase observed in the consortium (SaPA1 + SaBR2) (Figure 3). This coordinated increase in below- and above-ground fresh biomass suggests that inoculation promoted overall plant growth rather than reallocating resources to a single organ. A similar pattern has been reported previously, with endophytic inoculation increasing both shoot and root biomass (Zhang et al., 2013). This is biologically reasonable because a large root system can improve water and nutrient uptake, thereby supporting carbon assimilation and shoot development (Chen et al., 2014). The biomass responses were also associated with improved Zn acquisition. The changes in oxalic acid exudation followed trends similar to those of biomass increase and Zn accumulation, suggesting a root-mediated process in which endophyte-driven changes at the root interface may facilitate Zn mobilization and uptake (Chen et al., 2014). Overall, bacterial inoculation increased root and shoot biomass, supporting plant establishment and productivity. The accompanying improvement in root function may also have contributed to increased Zn uptake and internal distribution. Together, these responses support the potential application of endophytic bacteria as a microbe-assisted approach for yield improvement and Zn biofortification.

**Figure 4:**
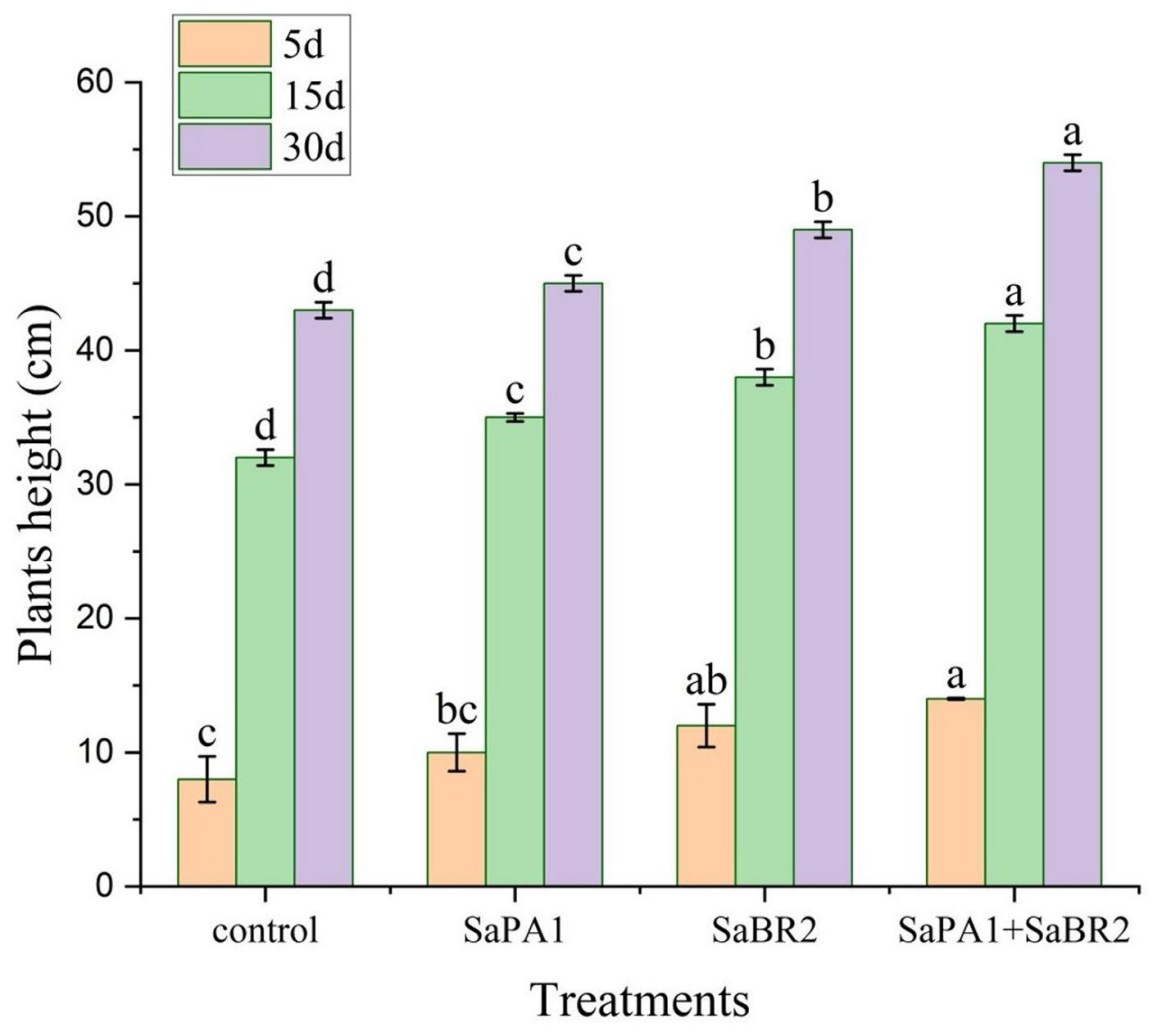
Changes in plant height at 5, 15, and 30 days under different treatments are displayed in a bar-graph. Different lowercase letters indicate significant differences among treatments at the same sampling time according to ANOVA (*p* < 0.05).

Our plant-height data (Figure 4) indicate that all inoculated treatments increased plant height relative to the control, with treatment differences becoming clearer as the plants matured. By day 30, the consortium (SaPA1 + SaBR2) produced the tallest plants (54 cm), followed by SaBR2 (49 cm), and SaPA1 (45 cm), and the control (43 cm) (Figure 4). This trend suggests that co-inoculation under hydroponic conditions may have promoted plant growth more effectively than single-strain inoculation, supporting the idea that strains combining can produce a stronger cumulative effect. Mechanistically, the observed growth pattern aligns with prior research, showing that endophytic inoculation can promote plant development through phytohormone-related effects, improved nutrient acquisition, and enhanced root-associated functioning (Liao et al., 2025). Similarly, Wang et al. (2014) reported that endophytic strains isolated from *Sedum alfredii* markedly enhanced plant growth under hydroponic conditions, supporting a clear growth-promoting effect in a nutrient-solution system. In the present study, the greater plant height observed under consortium inoculation may reflect improved root-associated functioning and nutrient acquisition, thereby supporting shoot elongation.

Root morphology is a crucial factor in plant nutrient uptake, because an extensive root system enhances the contact area between roots and the surrounding nutritional media. This study demonstrated that bacterial inoculation enhanced total root length and root surface area, suggesting that endophytic bacteria facilitated root system development (Table 1). The root images confirm this trend, as inoculated plants exhibited more extensive and denser root systems compared to the control. These structural enhancements may improve the root-solution interface, hence augmenting the plant’s ability to absorb water and nutrients. The most robust response in the consortium treatment indicates that the combination of bacterial strains may produce synergistic effects on root development. Microbial consortia often possess complementary plant-growth promoting traits, including the synthesis of phytohormones, nutrient solubilization, and alteration of rhizosphere conditions (Emami et al., 2019). These pathways may promote root extension and branching, hence enhancing root architecture. Prior research has similarly reported that microbes associated with plants enhance root growth and area, hence improving nutrient uptake efficiency (Chen et al., 2014). In the present study, this relationship was also evident at the treatment level; the consortium showed the largest total root length and root surface area, along with the highest Zn concentration and accumulation, whereas the control group showed the lowest values across these traits.

### 4.2 Improving Zn biofortification in wheat using individual and consortium bacterial inoculations

The endophytic bacterial strains selected for this study were originally isolated from *Sedum alfredii*, a well-characterized Zn-accumulating plant known for its strong capacity to absorb and accumulate Zn.

Our findings show that single-strain inoculation increased Zn levels, whereas the bacterial consortium produced the greatest increase in Zn concentration and accumulations in both roots and shoots, suggesting a synergistic effect on Zn uptake. Zn concentrations remained higher in roots than in shoots, across all treatments, indicating substantial Zn retention in root tissues, alongside enhanced uptake. This pattern aligns with prior endophyte-based studies, showing that bacterial inoculation can improve Zn uptake in certain plant systems by augmenting Zn availability near the roots and supporting plant Zn absorption (Chen et al., 2014). A similar trend has also been reported in rice inoculated with endophytes derived from *Sedum alfredii*, where Zn concentration and accumulation increased in both roots and shoots (Wang et al., 2014). Overall, these findings indicate that endophytic inoculation may improve Zn acquisition in specific plant–microbe combinations, although the extent and expression of this response are expected to vary among crop species and experimental conditions.

Specifically, endophytic inoculation has been associated with improvements in root traits that increase the root-soil contact interface, such as greater roots length, more root tips, and larger root surface area, thereby improving Zn acquisition. Consistent with this pattern, our data further show that the consortium increased total Zn uptake and allocation between roots and shoots in comparison to single-strain inoculations, suggesting a more coordinated “Zn capture and distribution” response in wheat. This interpretation is supported by Emami et al. (2019), who reported that “rhizospheric–endophytic mixed inoculants with multiple plant probiotic traits improved plant growth and micronutrient concentration. Similarly, Zhang et al. (2020) described plant probiotic consortia as bacterial assemblages that collaboratively promoted the plant growth and reduced the stress. In addition, improved root development and altered root exudation may have facilitated more efficient Zn uptake in plants. Our previous study showed that endophytic bacteria from *Sedum alfredii* exhibit plant-growth-promoting and Zn-solubilizing traits that support micronutrient uptake, while transporter-related responses, including TaZIP-mediated Zn transport, have been reported in wheat under Zn-related conditions (Li et al., 2025). Together, these findings support the potential of Zn-solubilizing bacteria as promising biofertilizers for enhancing agronomic Zn utilization (Singh et al., 2017). Overall, our findings suggest a consortium-driven enhancement in (i) Zn availability within the root zone and (ii) the uptake and handling capacity of plants through root-interface interactions, resulting in increased Zn retention in roots and its translocation to shoots.

### 4.3 Bacterial regulation of leaf gas exchange and photosynthetic efficacy

**Figure 5:**
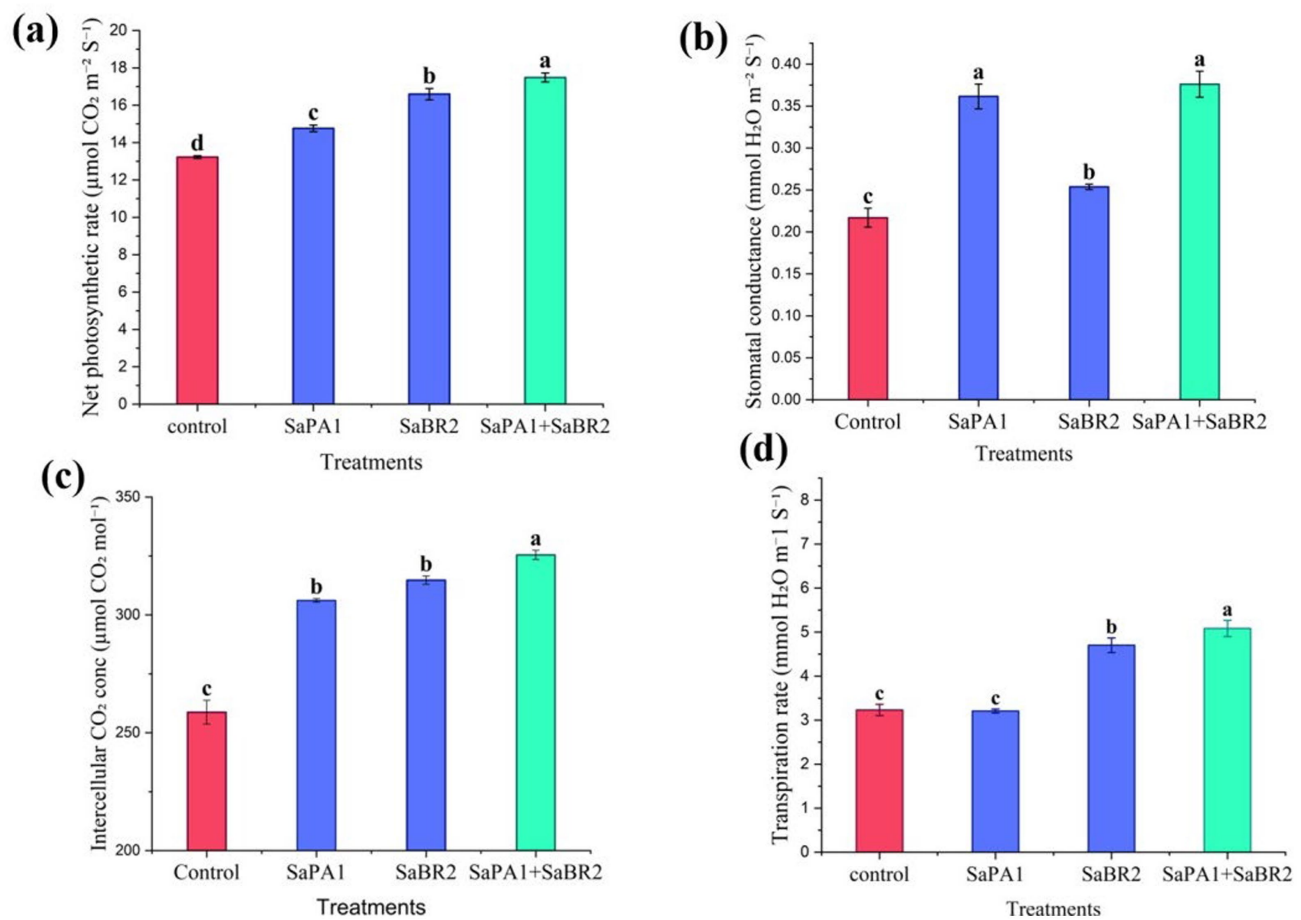
Effects of different bacterial treatments on leaf-gas exchange parameters in wheat leaves: **(a)** net photosynthetic rate, **(b)** stomatal conductance, **(c)** intercellular CO_2_ concentration, and **(d)** transpiration rate. Error bars denote standard deviation. Different lowercase letters indicate significant differences among treatments, as shown using ANOVA (*p* < 0.05).

Endophytic inoculation was associated with higher net photosynthetic rate (A), stomatal conductance (gsw), intercellular CO₂ concentration (Ci), and transpiration rate (Emm/E) (Figure 5). All measured photosynthetic metrics increased under bacterial treatments, particularly in the consortium, suggesting that inoculation may support greater CO₂ assimilation and more active gas-exchange relative to the control. These findings align with a previous study indicating that growth-promoting endophytic bacteria enhance photosynthetic efficiency in inoculated plants (Yuan et al., 2018). Physiologically, stomatal opening is essential for CO₂ entry during photosynthesis, while transpiration facilitates water transfer and enhances plant absorption. Transpiration also helps preserve tissue hydration and turgor, stabilize cellular structure, distribute inorganic ions, and eliminate excess heat produced during photosynthesis and oxidative metabolism (Li JiangHua et al., 2009). Prior research suggests that endophytic bacteria may augment chlorophyll-related functions and photosynthetic efficiency, potentially leading to enhanced plant growth (Yuan et al., 2018). The observed enhancement in photosynthetic efficiency may have contributed to greater carbon assimilation, which may have been translocated belowground and influenced root exudation, suggesting a possible physiological and functional connection between leaf-level carbon fixation and root-zone metabolic processes (Kaiser et al., 2015). In addition to supporting photosynthesis, these microorganisms may provide dual advantages for crop performance. By supporting gas exchange and the transpiration stream, they may contribute to more effective Zn uptake and its internal distribution within the plant. Simultaneously, elevated photosynthetic activity may increase carbon availability for growth, thereby supporting broader developmental improvement (Yuan et al., 2018). Thus, the observed responses align with the potential of endophytic inoculation as a microbe-assisted approach to enhance plant growth and nutrient uptake efficiency.

### 4.4 Root exudates

Root exudates consist of a complex array of compounds that facilitate nutrient mobilization and modify the rhizosphere (Jones et al., 1996). The combined evidence from PCA/OPLS-DA, volcano plots, and KEGG enrichment suggests that endophytic inoculation (SaPA1, SaBR2, and SaPA1 + SaBR2) reprogrammed wheat root exudation, generating stable, treatment-dependent metabolic states relevant to rhizosphere conditioning and Zn uptake efficiency. Accordingly, the clear separation of inoculated groups from the control in PCA and the directional discrimination in OPLS-DA are consistent with a regulated shift in exudate chemistry driven by bacterial colonization rather than random variation (Figures 2a–d). The volcano profiles indicate broad metabolomics remodeling, particularly in the consortium, which showed a more balanced regulation pattern (Figure 2e). KEGG analysis indicates that inoculation altered pathways linked to primary metabolism, particularly organic acid and amino acid metabolism, in addition to secondary and defense-related pathways, including phenylpropanoid/flavonoid and lipid metabolism. This pattern suggests that bacterial inoculation may have modified both the substrate availability for exudation and the metabolic processes related to root protection and plant–microbe interactions.

Primary metabolism supplies substrates and energy equivalents that shape exudate pools, whereas secondary metabolism provides bioactive molecules that stabilize root function and stress tolerance during microbial interaction. Secondary metabolites, such as flavonoids and phenolics, are important for stress tolerance and plant-microbe interactions, enhancing the stability of root function under changing rhizosphere conditions (Wu et al., 2025; Li et al., 2022). Furthermore, lignin synthesis resulting from phenylpropanoid metabolism facilitates structural fortification and the detoxification of reactive oxygen species (ROS) during microbial colonization (Barros and Dixon, 2020). Within this metabolic framework, Zn uptake may be promoted when exudate chemistry, especially organic acids, alters rhizosphere conditions. Organic acids released in root exudates may decrease rhizosphere pH and chelate Zn, thereby mobilizing less available Zn fractions and enhancing Zn availability for plant uptake. Previous studies, including Chen et al. (2014), have shown that low-molecular-weight organic acids, such as malate, citrate, and oxalate, play a crucial role in Zn solubilization and rhizosphere-mediated metal activation.

**Figure 6:**
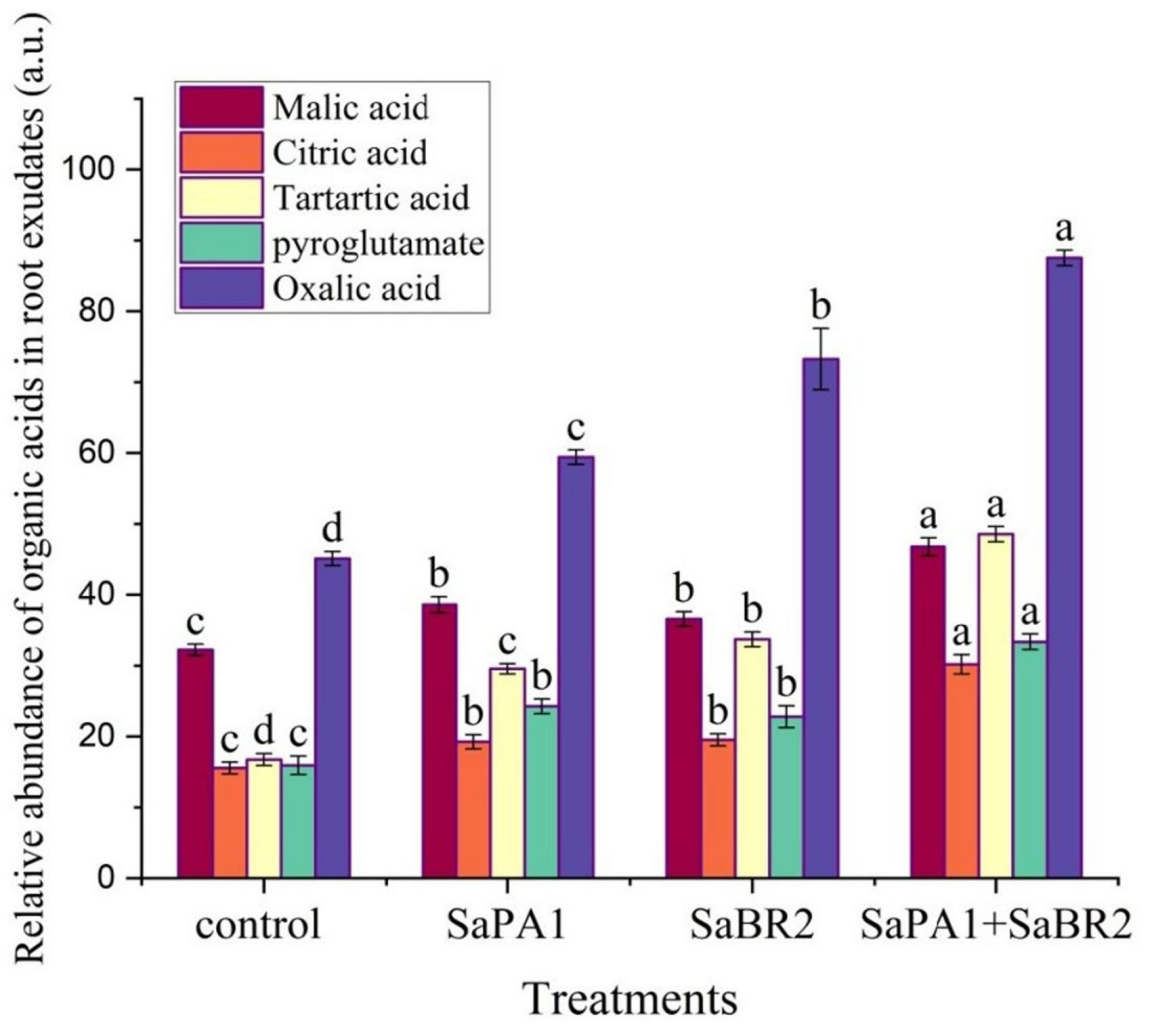
Relative abundance of major organic acids in wheat root exudates under different bacterial treatments. Error bars denote standard deviation (SD), and different lowercase indicates significant differences among treatments for organic acid at *p* < 0.05 (a.u., arbitrary units).

Notably, the enrichment of organic acid metabolism pathways indicates an increased metabolic flux towards the synthesis and secretion of organic acids. This pathway-level regulation is reflected in the heightened accumulation of organic acids identified in root exudates. The organic acid profile demonstrates a clear elevation between treatments, with the highest levels observed in the consortium, especially for oxalic acid, followed by malic, citric, and tartaric acids. This pattern suggests that organic acid synthesis was closely associated with treatment-dependent metabolite reprogramming (Chen et al., 2014). These findings align with previous studies indicating that organic acids containing two or more carboxylic groups can bind Zn and increase its mobility in the root environment, thereby contributing to greater metal bioavailability (Hoberg et al., 2005). In the present study, the elevated exudation observed under bacterial consortium inoculation was associated with the improved plant growth, root development, and higher Zn concentration and accumulation in both roots and shoots, suggesting a coordinated relationship between exudate remodeling and Zn acquisition. This pattern suggests a coordinated response in which metabolite reprogramming enhances organic acid exudation, potentially improving nutrient bioavailability, and supporting plant development and root function. These physiological enhancements can, in turn, influence plant metabolic activity and exudation patterns, hence strengthening the feedback link between metabolism and rhizosphere processes. This integrated response aligns with the function of root exudates in altering rhizosphere conditions via pH regulation and nutrient mobilization (Lu et al., 2013). Overall, these findings suggest that microbial inoculation reshaped root exudation in a way that likely contributed to rhizosphere conditioning and improved Zn uptake efficiency, thereby promoting wheat growth and Zn biofortification (see Figure 6).

Overall, our findings align with previous research indicating that endophytic bacteria can improve plant growth, root development, and Zn uptake through changes at the root interface and root exudation. However, the present study extends this existing literature by demonstrating, under controlled hydroponic conditions, that the SynCom of SaPA1 and SaBR2 produced the strongest combined effect on wheat growth, photosynthetic efficiency, Zn accumulation, and root exudate reprogramming. A particular strength of this work is the integration of physiological, nutritional, and metabolomic evidence within the same experimental framework, offering a more complete mechanistic basis for endophyte-assisted Zn biofortification in wheat. Nevertheless, we acknowledge that hydroponic conditions cannot entirely reproduce the complexity of soil composition, microbial interactions, and nutrient dynamics in agronomic environments. Consequently, the current results should be interpreted as mechanistic evidence obtained under controlled conditions, whereas our broader pot and field experiments, conducted with distinct strains and bacterial consortia across various soil types and Zn fertilizer applications, offer supplementary validation for the applicability of these findings to soil-based systems.

## 5 Conclusion

This hydroponic study showed that endophytic bacteria derived from *Sedum alfredii* can significantly improve wheat growth, physiological performance, and Zn nutrition under controlled soil-free nutrient solution environments. Inoculation with SaPA1, SaBR2, and especially the SynCom (SaPA1 + SaBR2) enhanced the observed responses, with the SynCom showing the strongest overall effect and highlighting the potential advantage of co-inoculation for Zn biofortification in wheat. Furthermore, non-targeted LC–MS profiling indicated that inoculation altered root exudation and reprogrammed essential metabolic pathways, establishing a reliable foundation for rhizosphere conditioning that enhances Zn acquisition. Overall, the results highlight that endophytic inoculation, particularly the SaPA1 + SaBR2 consortium, is an effective microbe-assisted strategy that enhances plant productivity while promoting Zn biofortification potential in wheat. The metabolomics results further suggested that bacterial inoculation reshaped root exudation and altered essential metabolic pathways associated with root-interface processes and Zn acquisition. Overall, these findings support the potential of endophytic inoculation, specifically the SaPA1 + SaBR2 consortium, as a promising microbe-assisted approach for enhancing wheat productivity and Zn biofortification. However, as this study was performed in hydroponic conditions, the results should be regarded as controlled mechanistic evidence. Their wider agronomic significance will be presented in our future manuscripts reporting the complementary pot and field experiments conducted under soil-based conditions.

## Data Availability

The original contributions presented in the study are included in the article/Supplementary material, further inquiries can be directed to the corresponding authors.
