# Supplementary material for: Co-inoculation of endophytic bacteria enhances hydroponic wheat performance and zinc biofortification through root exudates modulation
Source: Front Microbiol. 2026 Jul 10;17:1844753. doi: 10.3389/fmicb.2026.1844753 (PMC13396116; doi:10.3389/fmicb.2026.1844753)
Supplement: Supplementary file 1 [file Table_1.DOCX]

**Supplementary Data**


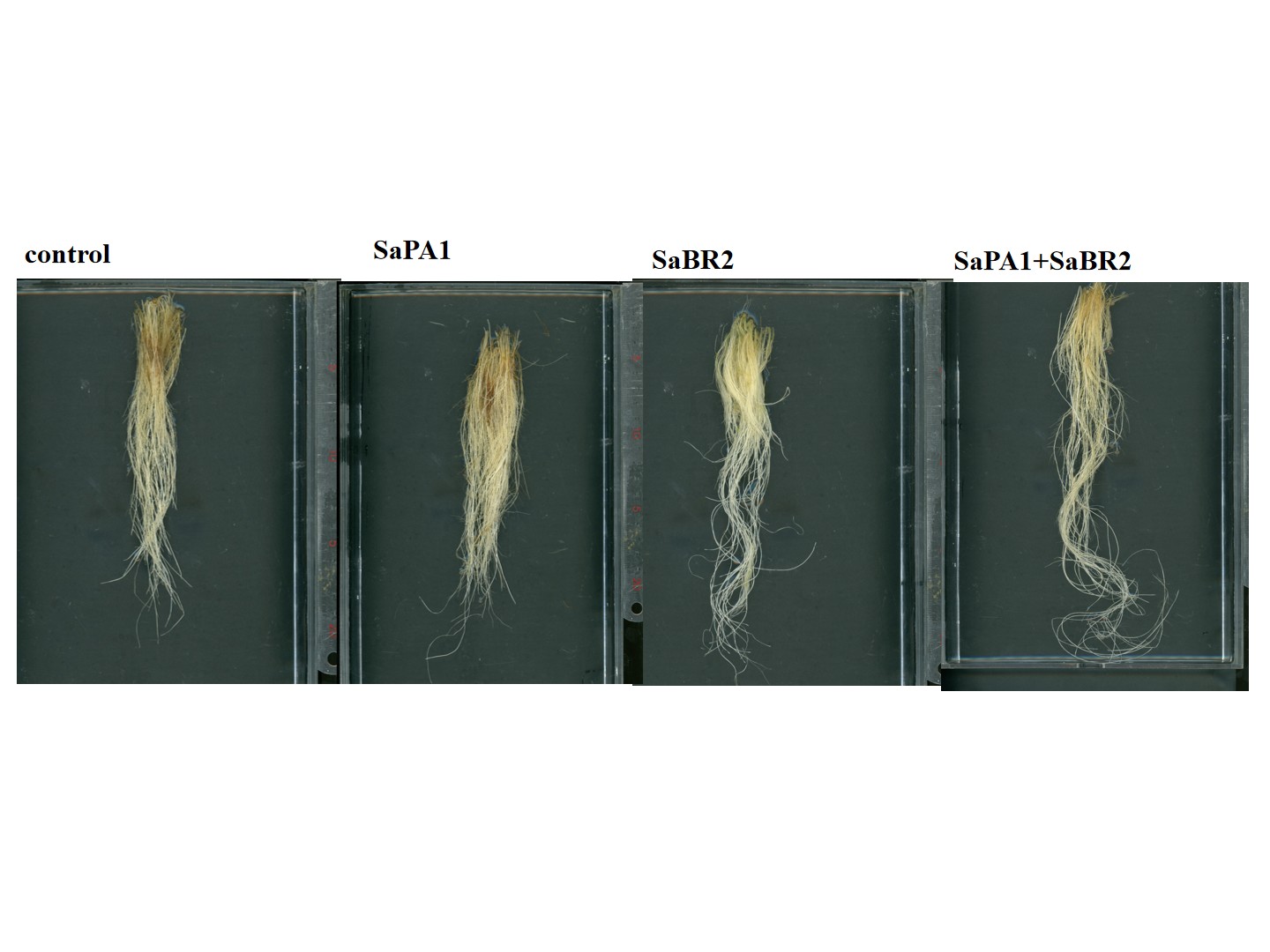


**Fig. 1: Root morphology of wheat plants inoculated with different bacterial treatments.**
